# Hospice Care in the Era of AI: Hospices' Views on Data‐Driven Tools to Support Live Discharge Decisions

**DOI:** 10.1111/jgs.70621

**Published:** 2026-08-03

**Authors:** Elizabeth A. Luth, Caitlin Brennan, Susan Hurley, Kira G. Sheldon, Yongkang Zhang

**Affiliations:** ^1^ Rutgers University New Brunswick New Jersey USA; ^2^ Care Dimensions Danvers Massachusetts USA; ^3^ New York University New York New York USA; ^4^ Weill Cornell Medicine New York New York USA

**Keywords:** AI, dementia, hospice live discharge, prediction tools

## Abstract

**Background:**

Live discharge occurs for 20% of hospice enrollees, resulting in loss of support and disruptive care transitions, with higher risk for patients with Alzheimer's disease and related dementias (ADRD). Little is understood about how data‐driven clinical decision support tools (e.g., predictive algorithms) might support decision making regarding live discharge. As hospices adopt value‐based care, identifying opportunities and challenges for data‐driven tools to predict and support live discharge holds great potential to support hospice patients and their caregivers.

**Methods:**

Semi‐structured interviews were conducted with 20 hospice leaders in clinical care, quality, and data science at seven non‐profit United States hospices. Four‐step rapid analysis and deductive approaches were used to summarize interview content in response to research questions and identify cross‐organizational themes.

**Results:**

Participants identified multilevel—individual and family, organizational, community, and system—challenges and facilitators to support patients following live discharge. Families dealing with ADRD face a heavier care burden but also access ADRD‐specific programs. Participants expressed strong interest in using predictive tools to identify patients at increased risk for live discharge and either support them to remain in hospice or facilitate robust discharge planning. Participants emphasized the importance of tool specification and clinical workflow integration to make predictive tools useful and impactful.

**Conclusions:**

Hospices face barriers to support hospice patients and caregivers experiencing burden and suboptimal outcomes following live discharge. Predictive modeling could be a potentially powerful tool to facilitate support for patients discharged alive, provided they are accurately specified and thoughtfully integrated into clinical workflows.

## Summary

- Key points
  ○ Hospice patients and organizations face individual‐, family‐, organizational‐, and community‐level challenges following live discharge. Patients living with ADRD and their families face unique challenges related to caregiving needs and burden and variable access to primary and specialty physicians and other supportive services.
  ○ Although not currently in use, hospices are interested in leveraging data‐driven tools to identify patients at risk for live discharge early and either (1) make a case for continued hospice enrollment or (2) provide additional support through the live discharge process. The former can be particularly relevant when ADRD is present.
  ○ To be useful, data‐driven tools must be trusted and perceived useful by clinicians, rely on accurate data, and be seamlessly incorporated into clinical workflows.
- Why does this paper matter?
  ○ This paper advances our understanding of multilevel factors hospices face in supporting patients discharged alive. It establishes hospices' interest in adopting data‐driven tools to support live discharge and outlines criteria for successful development and implementation of these tools.

## 1 Introduction

Approximately 20% of hospice patients disenroll before dying, experiencing what is called a live discharge [1]. Live discharge is an unintended consequence: individuals enroll in hospice with the expectation they are terminally ill with 6 months or less to live and will remain with hospice until they die [2]. However, patients can be discharged alive due to condition stabilization, which is initiated by the hospice agency, or from their own decision to pursue disease‐directed therapies (patient‐initiated discharge), which Medicare will not typically pay for simultaneously with comfort‐focused end‐of‐life care [3, 4]. Despite close monitoring of live discharge by the Centers for Medicare & Medicaid Services and public reporting of hospices' live discharge rates [5, 6], live discharge rates have remained in the 16%–20% range since 2010, rising annually since 2020 [1, 7, 8, 9]. Patients living with Alzheimer's disease and related dementias (ADRD) are at greater risk—about one in four experience a live discharge—most often due to hospice‐initiated discharge following long lengths of stay and stabilization, owing in part to difficult prognostication and slower disease progression [10].

Regardless of reason, live discharge is broadly understood to be a negative experience [11], resulting in loss of medication, access to durable medical equipment, and hospice services such as nursing and social work [12, 13]. Individuals discharged from hospice may experience burdensome care transitions, including multiple hospitalizations and hospice readmission [14]. Over half of patients discharged alive die within 6 months [15]. Hospitalization, hospice readmission, and death within 6 months indicate uninterrupted hospice care would have been appropriate for these individuals. Patients living with ADRD have higher odds of emergency department use and lower odds of readmission to hospice following live discharge [15]. Substantial support needs among persons living with ADRD after live discharge result in high caregiver burden and burnout [16]. Previous research on hospice live discharge practices indicates hospices often lack formal processes to ensure patient needs are met following discharge [17], particularly when the live discharge notice period is not sufficient to put adequate post‐discharge supports in place. Hospice clinicians describe live discharge as causing them moral distress and to feel they abandoned the patient and families [17, 18, 19]. Given variation in hospice live discharge experiences and potential negative impacts on patients, families, and clinicians, live discharge guidelines and robust discharge practices are needed [17, 20].

Predictive algorithms could be a clinical decision support tool to help hospice agencies identify patients at increased risk for live discharge and adverse outcomes after discharge, providing an early signal of a need for robust discharge planning. Several predictive algorithms based on administrative data, electronic health data, or other datasets are used to identify individuals at increased risk of mortality and who may be eligible for palliative or hospice care [21, 22]. Although these tools may be useful in clinical and care decision‐making at the end of life [23], to our knowledge, no predictive algorithms have been developed or deployed in decision‐making regarding hospice enrollees, such as supporting live discharge decisions and discharge planning. Existing research identifying predictors of live discharge and post‐discharge outcomes among hospice patients provides an important empirical foundation for developing more advanced predictive algorithms for this context [14, 24].

Development and implementation of predictive algorithms for hospice enrollees need to align with the existing efforts, procedures, and practices hospice agencies use to identify and support patients at risk of live discharge. However, little is known about the processes agencies currently employ to identify patients at heightened risk. Understanding this existing landscape is essential to ensuring predictive tools are feasible, acceptable, and designed to complement—rather than disrupt—current efforts to support live discharge decision‐making and post‐discharge planning. This study aims to address these gaps by answering the questions: (1) What are barriers and facilitators hospice agencies encounter in supporting individuals experiencing live discharge and their families? (2) What is the potential for data‐driven tools (e.g., predictive algorithms) in identifying individuals at increased risk of live discharge? (3) What issues need to be addressed for hospices to incorporate data‐driven tools into workflows? Given the increased risk for live discharge and subsequent poor outcomes, especially among individuals living with ADRD and their caregivers, we highlight issues specific to this patient population throughout the analysis.

## 2 Methods

### 2.1 Study Design and Sample

This study used purposive sampling to identify 20 hospice employees at seven not‐for‐profit hospice agencies serving individuals in 11 states to conduct semi‐structured interviews between November 2024 and July 2025. Consistent with the goal of purposive sampling [25], participants were chosen if their hospice agency role encompassed one or more of the following: clinical care oversight, clinical operations, quality control or improvement, and data science or technology.

Study team members relied first on their networks with hospice agencies and then used snowball sampling to identify additional hospice agencies and individuals within an agency to approach for participation. The study team invited participants by email to participate in a 1‐h video conference interview about managing live discharge and post‐discharge burdensome transitions, particularly patients with ADRD. Additional agencies and employees were recruited until thematic saturation was reached. Two members of the study team (EAL and KGS) conducted each interview. After obtaining oral informed consent, (KGS) conducted the interview and (EAL) took detailed notes, asking clarifying questions as needed. Interviews lasted 29–59 min (average: 47 min) and were audio recorded and transcribed using automated transcription. Transcripts were checked by study team members and identifying information removed. Participants received a $100 gift card for their time.

The study team (YZ, EAL) developed the interview guide based on the research objectives. Participants described their experience with hospice live discharge, leading reasons for live discharge at their agency, perspectives on challenges and initiatives supporting individuals experiencing live discharge, and current and potential utility of data‐driven tools to identify individuals at risk of live discharge, inform clinical care, and facilitate support for discharged patients. We aimed to identify information about what a predictive algorithm should include and predict and, if developed, how it should be formatted and implemented. We asked participants about the general and ADRD hospice populations (See interview guide in Supporting Information). Study procedures were approved by the [Rutgers University] Institutional Review Board. To protect participant confidentiality, only first name, last initial, and job title were collected. All study records were securely stored on password‐protected servers.

### 2.2 Data Analysis

We analyzed interview data using a four‐step rapid analysis approach, which is appropriate for studies like this with a short timeframe and limited resources [26]. This approach also supports our deductive analysis approach to answer our research questions. First, during each interview, (EAL) took detailed notes in a template based on the interview questions and tailored to capture key points, including illustrative quotes. Second, immediately following the interview, the template was reviewed, alongside the transcript, by (KGS) for accuracy and to identify additional key information not initially captured. Third, after all interviews at a hospice agency were conducted, (EAL and KGS) consolidated interview summaries for each hospice into a second template designed to capture broad themes and subthemes. Fourth, (EAL and KGS) identified cross‐cutting themes across hospice organizations. Mentions of ADRD‐specific needs and responses were noted at all stages of analysis.

## 3 Results

Participants included thirteen women and seven men, seven chief medical officers, six clinical operations leads, four quality/safety leads, and three data scientists or technology leads. At the time of their interview, participants worked at seven not‐for‐profit hospice agencies: two Northeastern, two Southern, two Western, and one Midwestern. The hospices served individuals in 11 states. One hospice operated in an urban setting, one rural, two urban/suburban, and three both urban and rural. Average daily hospice census ranged from 110 to 5800. Five hospices operated in one state, one in two states, and one in five states. No observed themes applied only to patients with ADRD. Some challenges and responses were more common among this population as noted below.

### 3.1 Question 1: Supporting Patients and Families Experiencing Live Discharge

#### 3.1.1 Challenges

**FIGURE 1:**
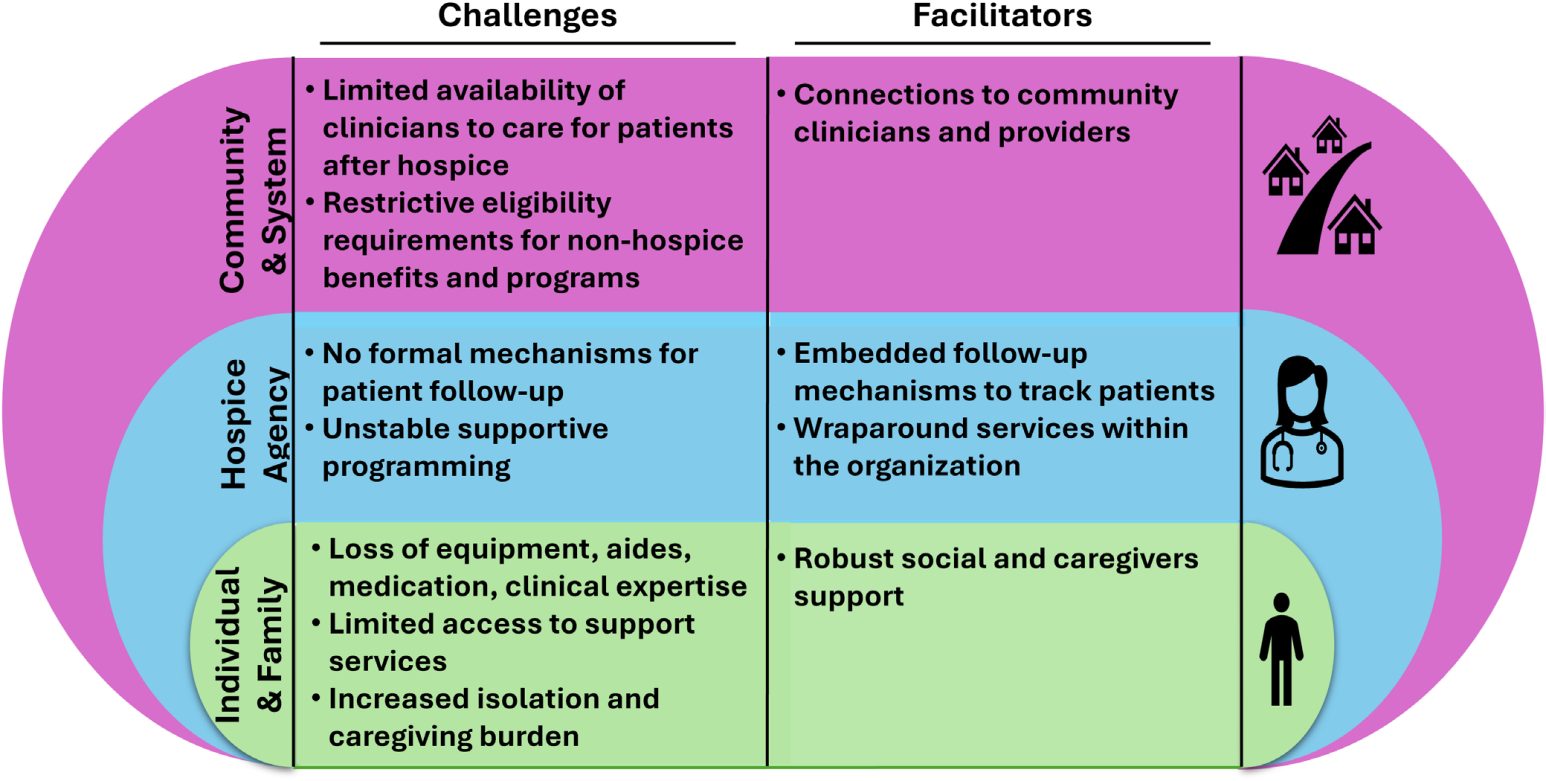
Multilevel challenges and facilitators to supporting patients experiencing hospice live discharge.

Participants indicated sometimes live discharge was inevitable, particularly when patients desired curative treatment. However, no participant characterized live discharge as desirable. Participants wanted patients and families to be supported following live discharge and identified several challenges patients and families often experience. Participant perspectives were based on experiences with patients and families, including upon hospice readmission, and/or knowledge from working closely with individuals providing hospice care. We grouped challenges into individual/family, hospice organization, and broader community/system levels. Participants emphasized their hospices worked to address challenges but felt limited in providing support once patients were not formally affiliated with the organization. We describe themes below and summarize in Figure 1. Example quotations in Table 1 illustrate themes.

**TABLE 1:** Multi‐level challenges to supporting patients and families through live discharge.

Level and themes	Exemplar quotations (participant type)
***Individual/Family Level***	
Loss of equipment, home health aides, medication (1.1)	I think in a lot of ways hospice is a safety net for these folks. We provide all of the care that they need and keep them in the home. And without hospice in place to help do that, …. They need to be going back to their primary [care physician]. They need to be going to the emergency rooms. It's harder for them to get equipment that they need or services that they need. (Quality)
Limited access to support services (1.2)	*So, if they are lower complexity, you know, in the Alzheimer's and dementia space, at that point and they're still able to live alone… But, you know, maybe they need help with meals, just finding those services and all of the different service areas And getting some of those services in some of those areas are very challenging*. (Data Science)
Loss of clinical expertise (1.3)	They're ending up with wounds because nobody's looking on a regular basis to see and doing prevention of wounds that are happening. (Quality)
Increased isolation (1.4)	But if you don't have that, you're estranged from your family or you outlived all your family or you never had children and your siblings are all dead and you never married and then you're just floating there, or maybe you even live alone. I mean, that's a hard thing to do effective discharge planning on. (Clinical Operations)
Increased caregiver burden (1.5)	*[For] dementia patients, the care burden is so extensive and the care needs are so significant, that I think for a lot of patients and families, it feels like a step back in terms of the care they're getting and maybe the outcomes as well… Most of the time on my discharges, it can be a hard pill for them to swallow*. (Clinical Operations)
***Hospice Organization Level***	
Lack of formal follow‐up mechanisms (1.6)	The problem with the monitoring part is once they're off service, we don't follow them anymore. So, monitoring is challenging… Once they're gone, they're gone. (Medical Director)
Unstable programming (1.7)	So, we have different palliative care programs in different places…now we've had some changes in funding…, it's been a little bit less ability to put people on palliative care than we had in the past. (Quality)
***Community/System Level***	
Limited availability of clinical providers to continue needed care (1.8)	The burden that we're noticing is pharmacy burden. Obviously, any palliative medications we gave the patient will have to be covered, that sort of thing after pain medications*…* And the kind of run‐away attitude that most of healthcare outside of hospice in a community would have. “We don't want to touch that,” sort of thing. (Medical Director)
	So, one is availability of primary care. Smaller communities, a lot of times difficult to find someone to take over their care. *And then even if you do, dealing with advanced dementia is something that a lot of our local primary care docs just aren't great at. Unfortunately. And we have no…. specialty providers. We have sort of one and a half neurologists … where I live, and so they're overwhelmed*. (Medical Director)
Restricted eligibility for non‐hospice benefits and programs (1.9)	“If they have Medicaid, it's great because you can just provide them home health aides using their Medicaid, but not every—certainly not even close, does every patient have Medicaid as a secondary. So, custodial care becomes a big problem off of hospice. How do you support a patient, ADL support in the home when it's not a Medicare benefit?*… If the Alzheimer's portion, which is really the burden of care to their caregiver, isn't a qualifier for Medicare skilled services just on its own, that's where coming off hospice—it'd be very frightening, I would think, for a family who's caring for someone with Alzheimer's*.” (Clinical Operations)
	*For the GUIDE model. They have to be—I don't think there's an age requirement on it, but it has to be one of X number ICD‐10 codes for Alzheimer's dementia, they've got to be within one of our service areas. So, just it's really more based on those criteria right now*. (Data Science)

*Note:* Italicized text indicates an example of a challenge for persons living with ADRD.

At the individual and family level, challenges related to immediate loss of hospice team support. This included instrumental support such as the loss of equipment, home health aides, and medication (Theme 1.1) and support services (Theme 1.2). Loss of clinical expertise and regular nurse visits can result in changes in patients' status (e.g., changes in eating, wound decompensation, early stages of a urinary tract infection) not being identified, resulting in emergency department visits and hospitalization and are indicators of appropriateness for hospice reenrollment (Theme 1.3). Many patients face increased isolation and inadequate caregiver support, particularly if residing alone (Theme 1.4). Family members also face increased social isolation and caregiver burden following live discharge, especially with ADRD, where patient personal care needs are extensive (Theme 1.5).

Hospice organizations face challenges establishing robust discharge plans to adequately support discharged patients and families. Participants cited the inability to monitor patients following live discharge: data on discharged patients is not typically collected by hospice agencies or may not be accessible to them (Theme 1.6). When hospices have programs to follow patients after live discharge, these programs may be unstable and dependent on variable funding sources, limiting their scope and size (Theme 1.7).

At community and system levels, discharged patients and families face challenges accessing clinical providers to support continuity of care (Theme 1.8). This includes limited access to prescribers with expertise to manage symptoms and pharmacies willing to dispense medications. For example, opioids, antipsychotics, and benzodiazepines are medications commonly used in hospice and palliative care. However, regulations responding to the opioid crisis and safety in long‐term care facilities result in limited access. Patients living with ADRD may face additional challenges in establishing care with a local physician with dementia expertise. Community resources or other programs may be limited geographically, especially in rural areas. Available programs may have strict eligibility requirements (Theme 1.9). For example, the GUIDE program, which supports persons living with ADRD, is not universally available and has eligibility criteria that some patients may not meet.

#### 3.1.2 Facilitators

Participants identified fewer facilitators available to support patients and families following live discharge. Figure 1 summarizes themes and Table 2 provides example quotations. At the individual level, they observed some families have knowledge, social connections, and financial means to provide ongoing support to their loved one following hospice discharge (Theme 2.1). At the hospice organization level, discharged patients could be referred to other services the organization provides—such as palliative care, physician house call programs, or GUIDE (for patients living with ADRD)—to continue support and engagement with patients and families (Theme 2.2). Some hospices had experience setting up systems to proactively follow up with discharged patients and for families to reengage with hospice when needed (Theme 2.3). These follow‐up and reengagement programs tended to be led by business development, rather than clinical, teams within hospice organizations. At the community and system levels, some hospices activated ongoing relationships with physicians in the local community, geriatrics departments at neighboring universities, skilled nursing and assisted living facilities, and community case management programs to ease discharged patients' transitions in care (Theme 2.4).

**TABLE 2:** Multi‐level facilitators to support patients and families through live discharge.

Level and themes	Exemplar quotations (participant type)
***Individual and Family Level***	
Robust social, caregiver support (2.1)	Some people are fortunate to have families who kind of rally around them. (Clinical Operations)
***Hospice Organization Level***	
Formal, hospice‐led follow‐up mechanisms (2.2)	We track formally if we can reconnect them with other services that we have*…*We do have primary care services, too, so we can be providers after. (Medical Director)
Wrap around services within the hospice (2.3)	I think mostly we just have this informal process with the Bridge Program where we know these patients are at high risk. Generally, the decision to come off of hospice is our decision… So, the families are usually very much in tune with us staying on board in some capacity. (Clinical Operations)
	*If we have to discharge someone for stability with dementia, we have this ability to refer them to either our palliative care team, if that's needed, or our GUIDE program, which offers structured support for that caregiver that can mimic*. (Medical Director)
***Community and System Level***	
Connections to community clinicians and providers (2.4)	There's a few people that I know in the community, because I was a primary care provider in this community… so, I have a pretty good understanding of, you know, how to communicate with the PCP. So, there's a few in the community that know enough that I can call them or email them and say, “Could you please take this patient on?” (Medical Director)
	We are affiliated with the Geriatrics clinic and the geriatrics program… Anytime we can identify skilled need, maybe it's med management, physical therapy, something along those lines, to kind of introduce [hospice] into the home so that it establishes a relationship and some trust. (Medical Director)

*Note:* Italicized text indicates an example of a challenge for persons living with ADRD.

### 3.2 Question 2: Potential for Data‐Driven Clinical Decision Support Tools to Identify Patients at Risk for Live Discharge

Most participants worked for hospices that did not use data‐driven clinical decision support tools relating to live discharge. When used, the tool reflected past trends rather than predicting future outcomes. Existing tools also presented issues with accessibility, availability, and the quality and synthesizability of data.

There are literally, I'm not kidding you, hundreds and hundreds of different insights and things we track. Too many, to the point where it's become actually overwhelming from a data to make decisions… So, we struggle more with having too many things that people have asked for over the years and trying to turn that data, information, knowledge to wisdom in a better funnel. Our funnel's way too big. (Data Science)

All participants indicated clinical decision support tools such as a predictive algorithm, if carefully engineered, could help identify patients at increased risk for live discharge in two ways. First, identifying individuals at increased risk could support early and robust discharge planning:

I mean literally using this tool could influence the plan of—it could at the very least help better the discharge, versus keeping them on. I mean, I can't always say that it's going to keep them on, but could it guide us to a better discharge plan? (Clinical Operations)

I think, you know, it provides useful clinical information for our decision‐making, but also, you know, because we're constantly balancing doing the right thing clinically, and also complying with the regulations. If we had, you know, a research‐validated tool where we could say, ‘Look, we applied this index, and it really shows that this patient has a greater than 50% chance of living more than the next six months.’ Then that, you know, sort of bolsters our decision‐making to have to discharge that person in a regulatory sense. (Medical Director)

Second, it could help identify individuals who may need to remain enrolled in hospice, for example, if they are likely to end up hospitalized following discharge, signaling where additional resources may be deployed to carefully document ongoing decline:

I would love to have some kind of a tool to justify when it was appropriate to keep those patients on hospice and when it is not. It's that million‐dollar question. Well, how long does he have? Well, I don't know. Weeks to months, months to years, days to weeks, hours to days. It just depends on what we're seeing, that sometimes even if you can't put your finger on it, you just know they're going in the wrong direction. It would be nice to have some kind of objective or clinical infrastructure to build a case on to keep these patients on hospice longer. (Clinical Operations)

### 3.3 Question 3: Successful Incorporation of Data‐Driven Tools Into Clinical Workflows

Participants provided varied feedback on important considerations for building a clinical decision support tool predicting hospice live discharge. They named a variety of potential users, including clinical managers, leadership, quality assurance, hospice team members, and potentially individuals in hospital palliative care and primary care who refer patients to hospice. They identified several individual data points to draw upon in building a tool, but emphasized inputs should rely on clean, consistently collected, clinically meaningful data. Participants also differed in what they wanted the tool to tell them in addition to live discharge risk, including short‐term mortality and hospitalization risk. Finally, they indicated it would be helpful to predict outcomes within short timeframes (e.g., days or weeks), although months may be useful in some cases.

#### 3.3.1 Implementation Challenges

**FIGURE 2:**
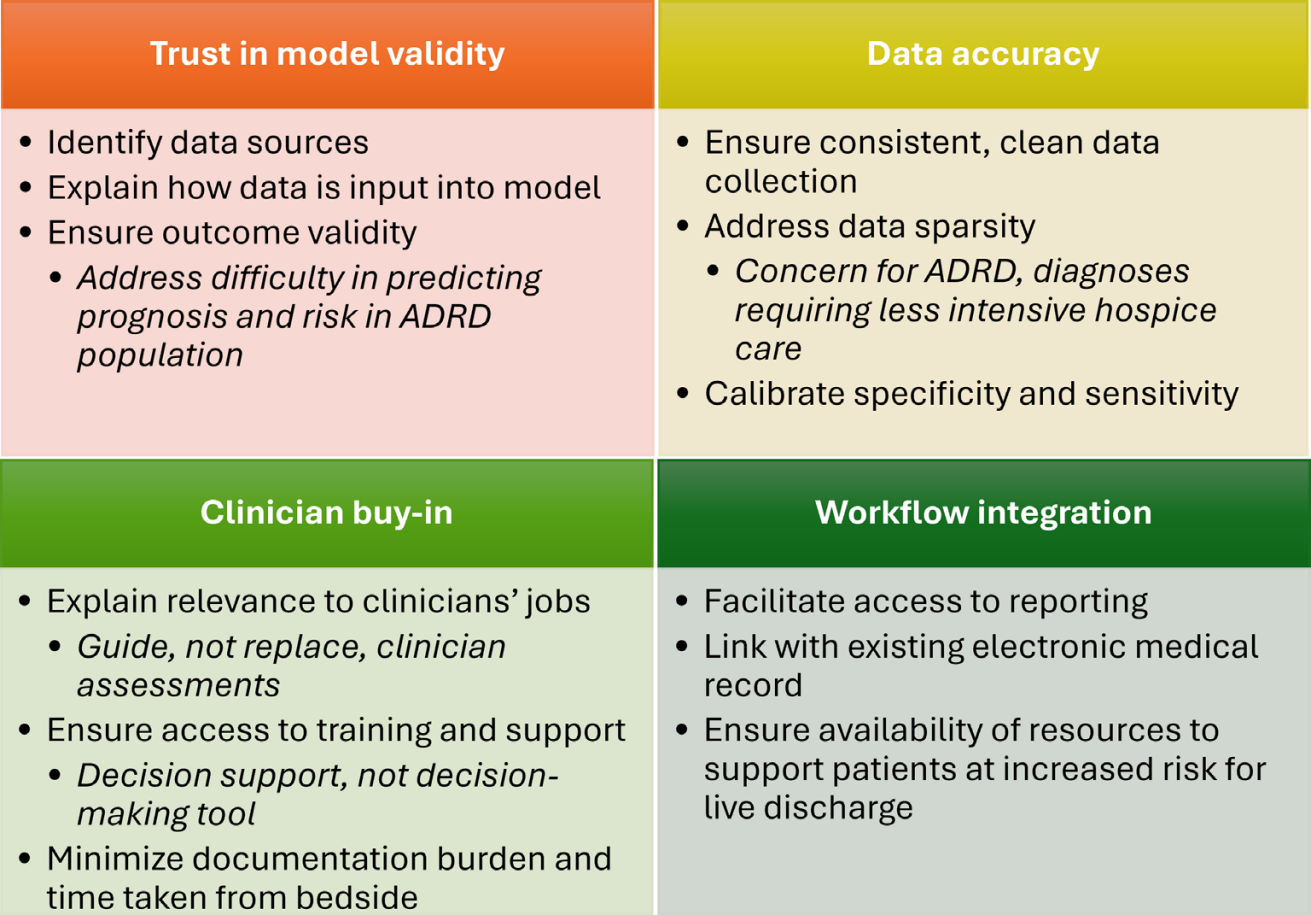
Elements hospice administrators identified for successful incorporation of data driven tools for live discharge into hospice clinical workflows.

Participants identified possible challenges to implementing predictive algorithms into clinical workflows, should one be developed and deployed (see Figure 2 for summary and Table 3 for exemplar quotations). Trust in model validity was the most consistently identified issue: it would be important users believed the model was accurately and consistently measuring what was intended (Theme 3.1). They expressed the importance of access to sufficient and consistent data, particularly with ADRD, when data sparsity is common, making it difficult to predict prognosis and trajectory with specificity and sensitivity (Theme 3.2). Participants identified challenges getting clinical staff to use the model, emphasizing the need to obtain buy‐in related to clinicians' understanding of its relevance, ensure clinical literacy in how to use it, provide training and ongoing support, and minimize burden from additional documentation to provide data (Theme 3.3). Finally, they indicated the importance of integrating the algorithm into existing clinical workflows, particularly the electronic medical record (Theme 3.4).

**TABLE 3:** Elements for successfully incorporating data‐driven tools into clinical workflows.

Theme	Example quotation
Trust in model validity (3.1)	Where is all the data coming from and how are we validating that it's accurate? How is it flowing into the predictive model? Are there people that are kind of falling off of that because they're not being seen? So is that going to sway all of it, being seen to begin with. And how do you validate the outcomes? (Quality)
Data accuracy (3.2)	Cases who have longer lengths of stay, again, related to dementia, is that they will have less service intensity. And so, it's a sampling bias. If you're not going to deliver enough visits, you're not going to pick up patterns to predict future outcomes. So again, you need to either deliver more visits or have another method of observing data that would predict those events, like care management calls, RPM [Remote Patient Monitoring]. (Data Science)
Clinician buy‐in (3.3)	Most staff don't know what a predictive model is…They often assume [it] is right 100% of the time… [they] need a bunch of clinical context to interpret what [it] means…[it] has to be decision support and not decision making, not prescriptive. (Data Science)
Workflow incorporation (3.4)	As a CMO [Chief Medical Officer] who gets pitched a lot, there's always this caution that you have. About some of these tools and algorithms that you really are able to build a clinical workflow that changes outcomes. And so… So I think… I would just say to you know put an equal focus over developing the tool onto the clinical flows and implementation science that will make it successful. (Medical Director)

## 4 Discussion

This study examined challenges and facilitators to hospices supporting the nearly 20% of patients experiencing a live discharge and the potential for data‐driven predictive algorithms as clinical decision support tools for supporting patients at increased risk of live discharge. Findings suggest increased burden and hardship following live discharge are common among hospice enrollees, and individuals with ADRD may face additional challenges such as considerable personal care needs. Based on direct experiences providing hospice care and/or knowledge gained working closely with hospice clinicians, participants identified individual and family challenges (e.g., loss of equipment and hospice support, increased patient and caregiver isolation) others documented [13, 16]. Participants provided additional context about how hospice agency and community and system level factors can facilitate or hamper post discharge outcomes. While clinical, caregiving, and social support may mitigate hardships individuals and families experience, access to these supports is often limited and dependent on community resources and eligibility criteria for supportive programs. Some hospices can mitigate these challenges and maintain ongoing contact with discharged patients, especially if they can refer patients to other programs (e.g., palliative care) their organization provides. Patients living with ADRD are at increased risk for hospice‐initiated live discharge and poor outcomes following hospice discharge, including difficulty finding clinicians with expertise in ADRD. Their families carry additional burden because of the high level of end‐of‐life care needs in this population. However, programs like GUIDE, when available, may provide support to mitigate post‐discharge difficulties.

Clinical, quality, and data science leadership at hospices expressed strong interest in using data‐driven clinical support tools to predict and manage live discharge, should one be developed. However, hospices have heterogeneous needs with respect to these tools. Any tool developed to support patients at increased risk for live discharge should consider this variability. Consistent with previous work identifying perceived variation in predictive models' accuracy for patients with different diagnoses and care use patterns [27], a “one size fits all” approach may not be appropriate. Tool development and integration may depend on hospice size, patient profiles, and hospice relationships with local health systems and community programs. Obtaining clinician trust in a predictive tool and integrating it into existing electronic medical record systems are also critical to successful adoption [28, 29].

Participants identified two potential functions of these tools. First, identifying individuals at increased risk for live discharge and subsequent transitions in care (e.g., hospitalization) may help hospice organizations allocate additional resources needed to make a compelling case for continued hospice enrollment, particularly with ADRD, where prognosis is difficult and trajectories are prolonged. Second, where live discharge is unavoidable or appropriate, early identification of patients who may experience it can provide additional time for comprehensive discharge planning to scaffold support for patients after hospice. Participants represented hospice leadership perspectives regarding utility of a predictive tool. However, identifying patients at risk for live discharge resulting in maintaining hospice enrollment or more robust discharge planning may also help address bedside clinicians' moral distress resulting from hospice discharge [17, 18, 19].

Most hospices operate in a data‐sparse environment. In 2022, 86% of hospices were freestanding agencies with limited interoperability [30], Their EHRs captured only patient information during the hospice stay without integrating longitudinal information prior to hospice enrollment from hospitals, nursing homes, home health, or other providers [31]. Yet pre‐hospice trajectories are strong predictors of live discharge and post‐discharge outcomes [14, 24], Administrative claims are not available to hospices in a timely manner for clinical decision support. Therefore, developing and implementing predictive algorithms requires comprehensive datasets and novel methods to link and synthesize fragmented data sources, recover patients' pre‐hospice trajectories, and generate timely, clinically actionable risk estimates that can be embedded into existing hospice discharge planning and care transition workflows.

## 5 Limitations

This study has limitations. All hospices included in our sample were non‐profit organizations. Their needs may differ from for‐profit hospices with different business models. Future work should consider the experiences and needs of for‐profit hospices. We used a purposive sample with participants with relevant experience to answer our research questions. Future work should confirm findings in a larger sample to ensure generalizability.

## 6 Conclusions

Patient and family outcomes following live hospice discharge are influenced by multilevel factors including personal and family financial resources, hospice organizations' additional services, and availability and accessibility of community‐based supportive programs. While ADRD involves additional care needs and subsequent family burden when hospice leaves, opportunities exist to support this population through disease‐specific programming. Provided they are properly specified, easily interpreted, and do not involve burdensome data collection for clinicians, predictive algorithms could be a useful clinical decision support tool to identify patients at increased risk of live discharge. They could provide hospices with an opportunity to build a case for ongoing enrollment or provide earlier, more comprehensive discharge planning to support patients and families, and timely hospice re‐enrollment as appropriate. However, even if well‐specified and implemented tools identify early patients at increased risk for live discharge, post‐discharge outcomes will depend on family caregiver support and availability and connection with non‐hospice services, whether provided by another part of the hospice organization or within the local community.

## Author Contributions

E.A.L., C.B., Y.Z. secured funding. E.A.L., C.B., S.H., Y.Z. created study design. E.A.L. and K.G.S. collected and analyzed data. E.A.L. drafted manuscript. All authors contributed significantly to editing manuscript for publication.

## Funding

This study was supported by a grant from the National Institutes of Health National Institute on Aging (R56 AG085541).

## Disclosure

This study was supported by a grant from the National Institutes of Health National Institute on Aging (R56 AG085541). The content of this manuscript is solely the responsibility of the authors and does not necessarily represent the official views of the National Institutes of Health.

## Conflicts of Interest

The authors declare no conflicts of interest.

## Supporting information


**Data S1:** Supporting Information.
