# Supplementary material for: Hospice Care in the Era of AI: Hospices' Views on Data‐Driven Tools to Support Live Discharge Decisions
Source: J Am Geriatr Soc. 2026 Aug 3;74(9):2659–68. doi: 10.1111/jgs.70621 (PMC13589668; doi:10.1111/jgs.70621)
Supplement: Supplementary file 1 [file JGS-74-2659-s001.pdf]

Interview Guide: Hospice Live Discharge and the Role of Data-Driven Tools

In this study, we are focused on understanding how we can better meet end-of-life (EoL) care needs and improve patient outcomes after hospice live discharge among older adults with Alzheimer's disease and related dementias (ADRD). We also want to understand the utility of developing and implementing data-driven analytics to do two things. First, to help identify patients at elevated risk for adverse outcomes such as burdensome care transitions. Second to facilitate care coordination among healthcare organizations and providers.

1. To get started, could you give me a big picture overview of what your roles and responsibilities are in your position?

2. What is your role in delivering hospice care? (may include administrative support of delivery)

3. In your work on hospice care delivery, have you had any focus on hospice live discharges, overall and ADRD specific? If yes, please describe.

4. What are the leading reasons for hospice live discharge among enrollees in your organization, overall and **ADRD specific**?

5. From your perspective, how does hospice live discharge impact health outcomes, quality of care, healthcare utilization, and mental status of hospice enrollees with ADRD and their caregivers?

6. Does your organization use any data-driven tools to facilitate decision making (e.g., hospice enrollment, identifying individuals at increased risk for live discharge)? If so, who developed these tools and who are using them (e.g., clinicians, administrators, or others)?

7. Does your organization have any established programs or initiatives that target hospice enrollees who were previously discharged alive from hospice, overall and **ADRD** specific?

8. Since **ADRD** patients may experience care transition across multiple care settings after hospice live discharge, has your organization established any collaborations or care coordination with other non-hospice providers and organizations to monitor patient outcomes after live discharge?

9. From your perspective, what are challenges to improving patient outcomes (e.g., reduce burdensome care transitions and reduce caregivers' stress and burden) after live discharge from hospice among older adults with **ADRD**?

10. Are you aware of the new CMS Hospice Care Index that includes quality measures related to hospice live discharge?
    a. *Summary of CMS policy:*

       In 2021, CMS added 4 measures related to hospice live discharge to their 10-item Hospice Care Index for hospice care quality. These 4 measures include early (i.e., ≤7 days of hospice enrollment) and late (i.e., >180 days of hospice enrollment) live discharges and 2 types of post-hospice burdensome discharge transition experiences. Type 1 burdensome transitions focus on individuals who are admitted to a hospital within 2 days following hospice live discharge, and then

readmitted to hospice within 2 days of hospital discharge. Type 2 burdensome transitions identify individuals who are hospitalized within 2 days after hospice live discharge and die while hospitalized.

11. From your perspective, what is the potential impact of the new CMS Hospice Care Index on hospice care delivery, particularly the way hospices discharge enrollees alive and manage post-discharge outcomes, overall and **ADRD** specific?

12. We learned from our previous projects that **ADRD** patients have a very uncertain trajectory at EoL, which makes their post-discharge trajectories and outcomes unpredictable. Do you think a prediction model that can predict the risk of adverse outcomes would be useful in clinical care delivery?

13. Is such a prediction model is developed, who are the potential users from your perspective? Will your organization be interested in testing it?

14. From your perspective, what are the potential challenges and barriers to use this kind of prediction tools to improve patient outcomes after hospice live discharge among older adults with **ADRD**?

15. Is there anything else you think would be important for us to know or think about as it relates to improve patient outcomes after hospice live discharge among older adults with **ADRD**?
